# Green synthesis of silver nanoparticles and evaluation of their effects on the *Porphyromonas gingivalis* bacterial biofilm formation

**DOI:** 10.1002/cre2.887

**Published:** 2024-05-26

**Authors:** Morad Hedayatipanah, Leila Gholami, Abbas Farmany, Mohammad Yusef Alikhani, Amirarsalan Hooshyarfard, Fahime Sadat Hashemiyan

**Affiliations:** ^1^ Department of Periodontics, Faculty of Dentistry Hamadan University of Medical Sciences Hamadan Iran; ^2^ Dental Implant Research Center, Faculty of Dentistry Hamadan University of Medical Sciences Hamadan Iran; ^3^ Department of Microbiology, Faculty of Medicine, Infection Disease Research Center Hamadan University of Medical Sciences Hamadan Iran; ^4^ Department of Periodontics, Dental Material Research Center, Faculty of Dentistry, Tehran Medical Sciences Islamic Azad University Tehran Iran; ^5^ Department of Periodontics, Faculty of Dentistry Qazvin University of Medical Sciences Qazvin Iran

**Keywords:** AgNPs, bacterial biofilm, *P. gingivalis*, propolis

## Abstract

**Objective:**

This study aimed to evaluate the impact of silver nanoparticles (AgNPs) synthesized from propolis on the formation of *Porphyromonas gingivalis* biofilms.

**Material and Methods:**

AgNPs were synthesized from propolis, and their inhibitory effect on *P. gingivalis* biofilm formation was assessed. Different concentrations of AgNPs (0.1%, 0.3%, and 0.5%) were tested to determine the dose‐dependent antibacterial activity.

**Results:**

The results of this study indicated that AgNPs exhibited an inhibitory effect on *P. gingivalis* biofilm formation. The antibacterial activity of AgNPs was dose‐dependent, with concentrations of 0.1%, 0.3%, and 0.5% showing effectiveness. Notably, the concentration of 0.5% demonstrated the most significant anti‐biofilm formation activity.

**Conclusion:**

The results of this study suggest that AgNPs synthesized from propolis have potential as an effective option for enhancing periodontal treatment outcomes. The inhibitory effect of AgNPs on *P. gingivalis* biofilm formation highlights their potential as alternative antimicrobial agents in the management of periodontal diseases.

## 1 INTRODUCTION

Periodontal disease is an inflammatory condition affecting the supportive tissues of teeth, caused by specific microorganisms. It leads to the progressive degradation of the periodontium through the host's inflammatory response (Hasan & Palmer, 2014). Epidemiological studies have revealed that severe periodontitis affects 5%−20% of the population, while a significant portion of adults experience mild to moderate periodontitis (Kassebaum et al., 2014). Among the known pathogens, *Porphyromonas gingivalis* is recognized as a crucial contributor to chronic periodontitis in humans. This bacterium has a bacillus‐shaped, asaccharolytic, immobile, gram‐negative, anaerobic nature, and belongs to the black‐pigmented family (Torkzaban et al., 2017, 2019). In medicine and dentistry, metal nanoparticles have gained attention due to their antibacterial properties (Hashemi et al., 2019; Song & Ge, 2019). Silver nanoparticles (AgNPs) are widely used, and known for their low toxicity and high biocompatibility with human cells (Corrêa et al., 2015; Giri et al., 2022). Propolis is a natural resin produced by bees, consisting of a mixture of bee saliva enzymes, pollen, and wax. This is a complex compound with numerous effects, including antibacterial, antiviral, antifungal, anti‐inflammatory, antioxidant, liver‐protective, anticancer, and immune‐regulating properties. It is often used to treat oral and gingival lesions (Šabanović et al., 2019). In dentistry, propolis is essential for preventing oral diseases such as caries and gingivitis. Numerous studies support the therapeutic effects of propolis in inhibiting microbial growth associated with caries and periodontal diseases. Additionally, this compound exhibits low toxicity and high biocompatibility (Freires et al., 2016; Libério et al., 2009). In a controlled clinical trial that examined the effect of topically administered propolis and curry leaves (a plant product) in the periodontal pockets of patients with periodontitis, it was found that treatment with propolis significantly reduced CAL, PPD, and the amount of *P. gingivalis* in GCF (Nakao et al., 2020). Moreover, the effect of propolis extract on inhibiting alveolar bone resorption in rats with experimentally induced periodontitis showed a significant reduction in the expression level of inflammatory cytokines in gingival tissue and alveolar bone resorption in the group treated with propolis extract (Sung et al., 2019). The antibacterial effect of ethanolic extract of propolis (EEP) on *P. gingivalis* bacteria demonstrated that EEP‐derived drugs could be effective in treating chronic periodontitis (Yoshimasu et al., 2018). Green‐synthesized AgNPs using propolis have exhibited strong antibacterial effects against both anaerobic and aerobic pathogenic oral bacteria (Lu et al., 2013). Examination of subgingival irrigation with propolis extract on teeth with chronic periodontitis in terms of clinical and microbiological parameters showed a significant reduction in the total number of anaerobic bacteria (Coutinho, 2012). Adding propolis to toothpaste has also demonstrated analgesic and plaque‐reducing effects (Kamburoğlu & Özen, 2011; Skaba et al., 2013). Geographically, different propolis samples have varying chemical compositions that directly impact their antioxidant properties. For instance, Russian and Italian ethanolic extracts of propolis exhibit a similar antioxidant effect due to their shared polyphenol composition, while Brazilian propolis has a comparatively lower antioxidant effect due to its lower polyphenol content (Fabris et al., 2013). Research on the properties of Iranian propolis has been limited and incomplete. The use of propolis is based on knowledge of its therapeutic properties, aiming to add economic value to raw propolis and develop innovative new drugs (Lobo et al., 2014; Zabaiou et al., 2017). Furthermore, it has been shown that the antimicrobial properties of AgNPs can be utilized as an alternative solution to reduce bacterial adhesion and prevent biofilm formation (Sivolella et al., 2012). Green‐synthesized AgNPs have a wide range of pharmaceutical and biological potential, including antiviral, antibacterial, antifungal, antiparasitic, antioxidant, anticoagulant, and more (Habeeb Rahuman et al., 2022). The antimicrobial activity of AgNPs on *P. gingivalis* bacteria has been reported (Halkai et al., 2017; Holden et al., 2016; Nakajima et al., 2016; Sundaram et al., 2019). In recent years, the growing emphasis on biocompatibility, low toxicity, and environmental friendliness has propelled the popularity of biosynthesis or green synthesis of nanoparticles. The utilization of natural materials like fungi (Honary et al., 2013), bacteria (Truong et al., 2022), algae (Chugh et al., 2021), and plant extracts (Firoz et al., 2022; Habibipour et al., 2019; Varadharaj et al., 2020) for AgNPs synthesis presents several advantages, including reduced energy consumption, manageable technology prerequisites, and the absence of harmful chemicals (Khan et al., 2022). Biological methods are inherently more eco‐friendly than their chemical and physical counterparts, primarily due to factors like the avoidance of hazardous byproducts, the utilization of biological entities as reducing agents, and their lower energy demands. While microbial species have demonstrated their potential in efficiently producing metallic AgNPs, there is still a need for expertise to comprehensively grasp and regulate the reduction process. Moreover, the challenges associated with maintaining a stable culture medium and optimizing conditions such as ideal pH, feasible temperature ranges, and salinity of the culture and reaction mixture underscore the complexities involved in scaling up these techniques for industrial use. Furthermore, in the case of plants and a selected few other biological species, including algae, certain natural chemical compounds present in the extracts serve the dual role of reducing and capping agents. This eliminates the requirement for toxic chemicals to function as capping agents. The approach of synthesizing metallic AgNPs using plant‐derived extracts, which can be sourced from leaves, roots, or stems, represents an eco‐friendly, uncomplicated method that encounters minimal economic and environmental barriers (Firoz et al., 2022; Sabri et al., 2016). It appears that the nanoparticle synthesis method using propolis is cost‐effective, biocompatible, and a simple alternative to conventional synthesis methods. Despite AgNPs being synthesized using propolis, no study has been conducted on their production from Iranian propolis or their effects on periodontal pathogens. Therefore, this study aims to investigate the effect of green‐synthesized AgNPs using propolis on the formation of the *P. gingivalis* bacterial biofilm, which plays a crucial role in the development and progression of periodontal disease.

## 2 MATERIALS AND METHODS

### 2.1 Bacteria cultivation under anaerobic conditions

The bacterium used in this study was *P. gingivalis* strain IR‐TUMS/BPG5 (GenBank: KX108929.1), obtained from the Tehran University of Medical Sciences. The purchased bacterium was in a lipophilized form, so the bacterial powder was diluted with sterile physiological serum. The bacteria were cultured on BHI agar (Brain Heart Infusion) (Merck), which was enriched with yeast extract (5 mg/L), vitamin K (1 mg/L), Hemin (5 mg/L), and sheep blood (5%) (Rafiei et al., 2018).

The culture plates were incubated in an anaerobic environment with CO_2_ (5%), H_2_ (10%), and N_2_ (58%) at 37°C for 48 h. To prepare the bacterial suspension for the experiments, *P. gingivalis* colonies from the BHI agar plates were transferred to test tubes containing BHI broth (Merck). The broth was further enriched with Hemin (1 mg/mL) and 5% horse serum. These test tubes were then incubated at a temperature ranging from 35°C to 37°C for 1−2 h (Seers et al., 2021).

### 2.2 Adjustment of optical absorption of *P. gingivalis* bacterial suspension

A sterile tube containing the bacterial suspension was placed in an Elisa reader (A&E lab/AE/S60/AU) to determine the bacterial concentration. The optical absorption of the suspension was read at the wavelength of 600 nm. The optical absorption of the suspension was adjusted from 0.08 to 0.11 to a turbidity equivalent to the standard half McFarland (CFU/mL) of 1.5 × 10^8^ bacteria (Dillen et al., 2006).

### 2.3 Synthesis of AgNPs

AgNPs were produced using propolis. To prepare the propolis, the initial step involved washing the sample with water, followed by drying it in an oven at 50°C for 48 h. For the hydroalcoholic extract, 50 g of the samples were immersed in 500 mL of 70% alcohol at room temperature for 72 h. The extract was then separated using the filtration method with a 40 µ Whatman filter paper. To completely separate the finely powdered particles suspended in the prepared extract, a centrifuge was employed. To synthesize the AgNPs, a solution of silver nitrate with a specific concentration was prepared. After adjusting the pH to 9.5, the prepared extract was gradually added to the silver nitrate solution. The mixture was then heated to 70°C and stirred for 2 h. The change in the solution's color to a bluish‐gray indicated the successful synthesis of AgNPs. The resulting precipitate was washed with double distilled water and subjected to centrifugation at 4000 rpm using a centrifuge (Behdad) (Dillen et al., 2006).

### 2.4 Nanoparticles characterization

The synthesized nanoparticles were characterized by using the following methods: XRD (X‐ray diffraction): To investigate the crystal structure of the nanoparticles, a Panalytical Xpert PRO X‐Ray Diffractometer (Panalytical) model Xpert Pro MPD with a wavelength of 1.5405 and power of 40 kV/30 mA was employed. TEM (Transmission electron microscope): An electron microscope was utilized to examine the surface morphology and size of the nanoparticles.

### 2.5 Treatment of microbial biofilm with AgNPs

The plates were divided into experimental and control groups. The control group did not receive AgNPs in the bacterial suspension. In the experimental group, the effect of green synthesized AgNPs on the inhibition of bacterial biofilm formation was evaluated. For this purpose, a bacterial suspension was prepared, and AgNPs with concentrations of 0.1%, 0.3%, and 0.5% were added to the suspension. The samples were incubated at 37°C for 24 h. Then, the formation of bacterial biofilm at OD (optical density) of 570 nm was read using the microtiter plate method and Elisa reader (Singhal et al., 2021).

### 2.6 Comparison between test and control group

The data from the two groups were compared, and the impact of AgNPs on bacterial biofilm formation was investigated. In a nutshell, 100 μL of a *P. gingivalis* microbial suspension with turbidity equivalent to half a McFarland was transferred to a 96‐well microplate. Subsequently, 100 μL of the studied AgNPs groups with concentrations of 0.1%, 0.3%, and 0.5% were introduced into the suspension. The microplate was then incubated for 48 h under anaerobic conditions at 37°C. After this incubation period, the wells of the 96‐well microplate were rinsed with saline phosphate buffer to eliminate bacteria in the planktonic phase. Wells that did not receive AgNPs served as the negative control group (Singhal et al., 2021).

### 2.7 Biofilm formation

The 96‐well microplate wells were rinsed with a saline phosphate buffer, and the microplate was left to air dry to measure the amount of microbial biofilm using a colorimetric method. The microbial biofilm formed at the bottom of the wells was stained by adding 200 μL of 0.1% crystal violet for 15 min. The plate was washed and dried three times with phosphate saline buffer to remove any excess dye. After that, 100 μL of 95% ethanol was added and left for 15 min, then the supernatant was gently removed and allowed to dry. Finally, 200 μL of 33% acetic acid was added to the wells, and an Elisa reader was utilized to measure the OD of each well at 570 nm.

### 2.8 Statistical analysis

The statistical analysis of the data involved the use of several tests, including the one‐way analysis of variance (ANOVA), independent *t*‐tests, and Tukey's range test. Tukey's test, also known as Tukey's Honestly Significant Difference test, was specifically employed to determine individual means and was particularly suitable for comparing more than two means. In contrast, the *t*‐test was used for comparisons involving two means. Tukey's test involved conducting pairwise comparisons of all means to identify significant differences. To determine the normality of the data, the Shapiro−Wilk test was employed. It is important to note that a significance level of 0.05 was considered for all statistical tests (Hazra & Gogtay, 2016). The statistical analyses were performed using SPSS version 23, a widely used software package for statistical analysis.

## 3 RESULTS AND DISCUSSION

### 3.1 Effect of AgNPs on *P. gingivalis* biofilm formation

The result of the evaluation of the effect of different concentrations of AgNPs on the *P. gingivalis* bacterial biofilm formation is presented in Table 1.

**Table 1:** Standard deviation, minimum, and maximum biofilm formation of *Porphyromonas gingivalis* bacteria (*n* = 3).

Variable	Mean	Maximum	Minimum	Variance	Standard deviation
Control	3.20	3.28	3.14	0.005	0.07
AgNPs (0.1%)	2.62	2.77	2.40	0.04	0.19
AgNPs (0.3%)	2.04	2.11	1.95	0.006	0.082
AgNPs (0.5%)	1.21	1.35	1.10	0.07	0.13

Abbreviation: AgNPs, silver nanoparticles.

**Figure 1:**
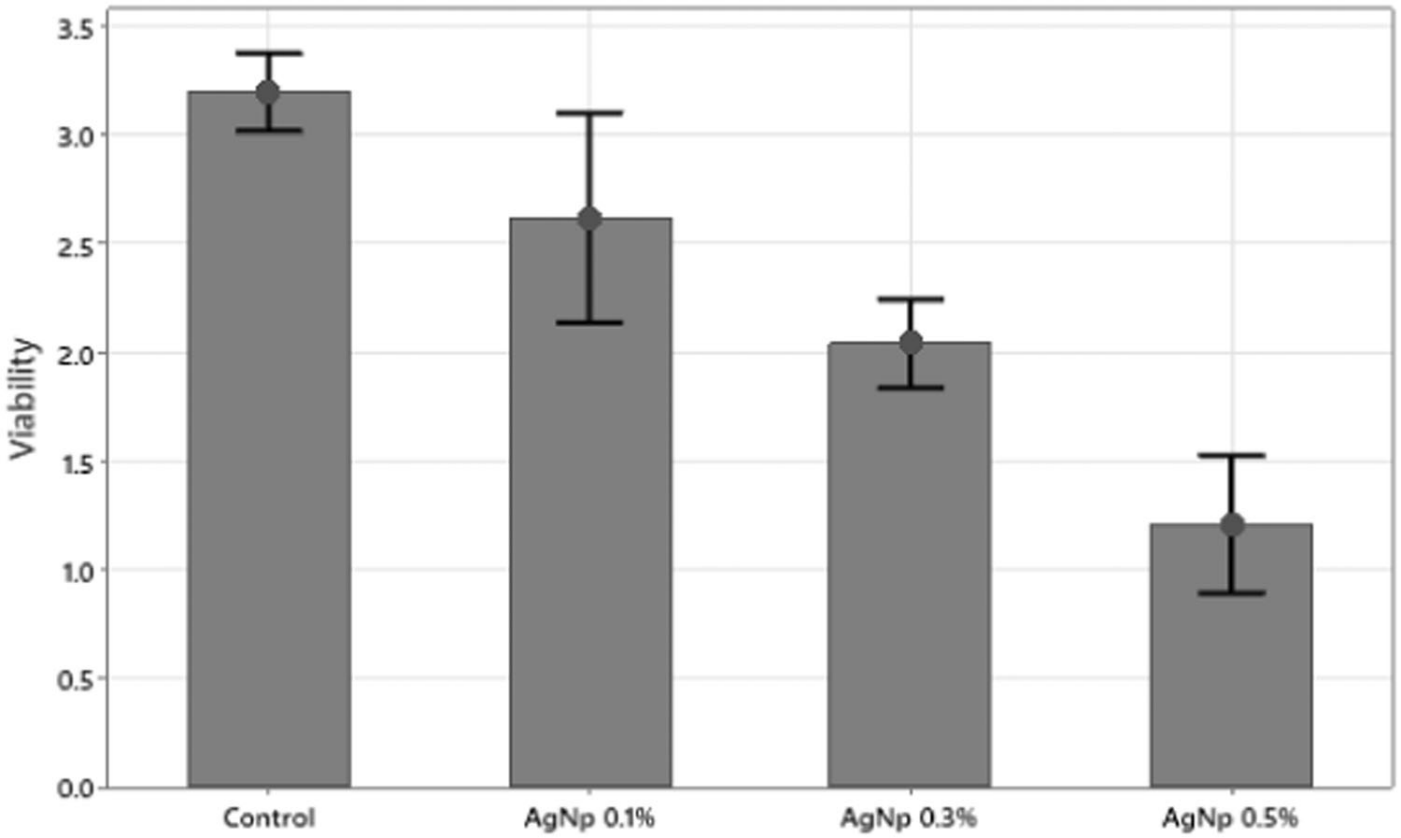
*Porphyromonas gingivalis* bacterial viability (%) in the presence of different concentrations of AgNPs. AgNPs, silver nanoparticles.

Figure 1, displays the data with an I‐shape representing the maximum and minimum values along with the average for each data group at the top of each bar. The graph in Figure 1 illustrates that the viability of *P. gingivalis* bacteria decreases as AgNPs concentration increases. Further examination through a statistical test is needed to determine if there is a significant difference between the groups (Table 1).

As there are more than two groups in the experiment, one‐way ANOVA is used to examine differences. One‐way ANOVA demonstrates if there is any significant difference between groups. The result of one‐way ANOVA shows that there was a significant difference in the control and different concentrations of AgNPs (Table 2). There were significant differences between *P. gingivalis* biofilm formation (*p* < .001).

**Table 2:** Mean *Porphyromonas gingivalis* bacteria biofilm formation (OD) compared to the control group (one‐way variance analysis).

	Average square error (Adj. MS)	Total modified squares (Adj. SS)	Degree of freedom (*DF*)	*F*‐value	*p* Value
AgNPs	2.16	6.49	3	130.95	.001
Error	0.016	0.13	8		
Total		6.62	11		

Abbreviation: AgNPs, silver nanoparticles.

After demonstrating significant differences between groups by one‐way ANOVA, it should be tested if all concentrations of AgNPs reduced *P. gingivalis* bacteria biofilm compared to the control group. So, the two‐sample *t*‐test is used to compare each group to the control group. The results showed that AgNPs reduce the microbial biofilm formation rate compared to the control group. As presented in Table 3, the results of the independent *t*‐test show that concentrations of 0.1%, 0.3%, and 0.5% of AgNPs can significantly reduce the *P. gingivalis* bacteria biofilm formation compared to the control group (*p* < .05). In other words, the use of AgNPs reduces the residual amount of the bacterial *P. gingivalis* biofilm.

**Table 3:** Two‐by‐two comparison of mean reduction of biofilm formation (OD) of *Porphyromonas gingivalis* bacteria compared to the control group treated with different concentrations of AgNPs using independent *t*‐test.

Variable hypothesis	Standard error	Confidence interval (95%)	*p* Value
Control—AgNPs (0.1%)	0.23	0.58	<.02
Control—AgNPs (0.3%)	1.01	1.16	<.001
Control—AgNPs (0.5%)	1.79	1.99	<.001

Abbreviation: AgNPs, silver nanoparticles.

The two‐sample *t*‐test can only examine the difference between two groups, and it cannot be used iteratively as the error would be accumulative, and its results would be unreliable. Tukey's post hoc was applied to rank groups based on the *P. gingivalis* biofilm. As shown in Table 4, there is a significant difference between all test groups, and the comparison of each test group with the control group was significant (*p* < .05). Increasing the percentage of AgNPs has a significant effect on the reduction of *P. gingivalis* bacterial biofilm formation and is less than the control group.

**Table 4:** Two‐by‐two comparisons of the mean reduction of biofilm formation (OD) of *Porphyromonas gingivalis* in three groups of AgNPs with concentrations 0.1%, 0.3%, and 0.5% using Tukey post hoc test.

Paired comparison of samples	Mean difference	Standard error	Confidence interval (95%)	*T*‐value	*p* Value
AgNPs (0.1%)—Control	−0.58	0.105	(−0.24 to −0.92)	−5.53	.002
AgNPs (0.3%)—Control	−1.16	0.105	(−0.82 to −1.50)	−11.05	.001
AgNPs (0.5%)—Control	−1.99	0.105	(−1.65 to −2.33)	−18.96	.001
AgNPs (0.3%)—AgNPs (0.1%)	−0.58	0.105	(−0.24 to −0.92)	−5.53	.002
AgNPs (0.5%)—AgNPs (0.1%)	−1.41	0.105	(−1.07 to −1.75)	−13.43	.001
AgNPs (0.5%)—AgNPs (0.3%)	−0.83	0.105	(−0.49 to −1.17)	−7.91	.001

Abbreviation: AgNPs, silver nanoparticles.

In this study, the mean reduction in the biofilm formation was also investigated, and the results are presented in Table 5.

**Table 5:** Comparison of the mean reduction in biofilm formation (OD) of *Porphyromonas gingivalis* bacteria in three groups of AgNPs with concentrations 0.1%, 0.3%, and 0.5% using Tukey sequential test (*n* = 3).

Factor	Mean	Groups			
Control	3.20	A			
AgNPs (0.1%)	2.62		B		
AgNPs (0.3%	2.4			C	
AgNPs (0.5%	1.21				D

Abbreviation: AgNPs, silver nanoparticles.

### 3.2 Characterization of AgNPs

**Figure 2:**
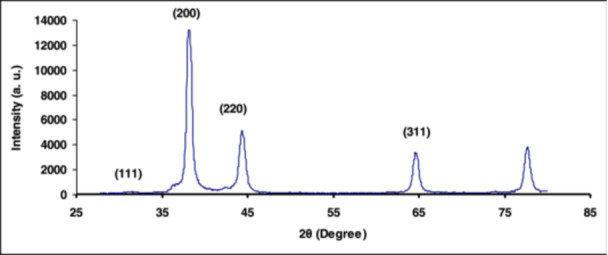
X‐ray diffraction pattern of AgNPs. AgNPs, silver nanoparticles.

Figure 2 shows the XRD pattern of the synthesized AgNPs. The main peaks recorded at 2θ in the areas of 31.7, 38.2, 44.4, and 64.8 are related to Miller indices (111), (200), (220), and (311), respectively (Habibipour et al., 2019).

**Figure 3:**
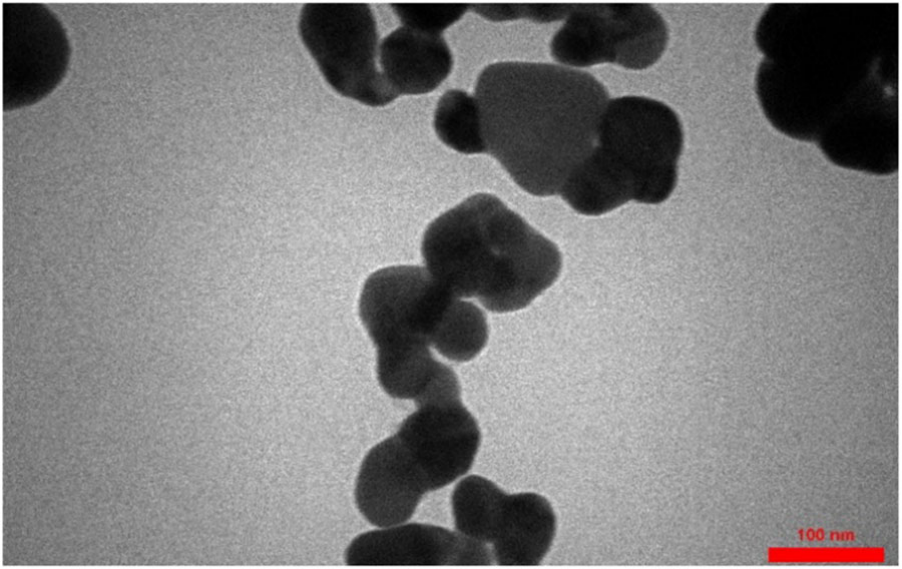
TEM image of AgNPs (scale bar 100 nm). AgNPs, silver nanoparticles; TEM, transmission electron microscope.

Figure 3 shows a TEM image of synthesized AgNPs. TEM imaging results show that colloidal AgNPs were obtained with a diameter of less than 100 nm during the synthesis process (Habibipour et al., 2019).

The biological activity of inorganic nanoparticles may be influenced by some physicochemical factors, including size distribution, morphology, surface charge, surface chemistry, capping agents, and so forth (Barabadi et al., 2020). There are a lot of reports that demonstrate the effect of the synthesis method on the size, morphology, dose, exposure time, and cytotoxicity of AgNPs (Barabadi et al., 2020).

### 3.3 Comparing results with previous studies

AgNPs are well‐known nanomaterials with inhibitory and antibacterial effects. Silver ions released from these nanoparticles can bind to glucose, oxygen, or nitrogen in the bacterial cell wall biomolecules, disrupting membrane integrity and leading to cell death (Barabadi et al., 2021; Casemiro et al., 2008; Talank et al., 2022). At this time, various methods exist for synthesizing nanoparticles, but green methods have replaced physical and chemical ones due to their reduced environmental impact. Green methods involve the use of or production of environmentally safer materials (Viuda‐Martos et al., 2008). AgNPs target inhibitory components in the bacterium's outer membrane, causing the release of molecules like lipopolysaccharides and purines from the cytoplasm. They also inactivate bacterial enzymes upon penetrating the bacterial cell, leading to cell death through the production of hydrogen peroxide (Choma & Grzelak, 2011). Notably, silver ions significantly increase the production of reactive oxygen species, including superoxide anion radicals, inducing bactericidal effects (Barabadi et al., 2021; Talank et al., 2022). This oxidative stress occurs at the cellular and molecular levels, affecting organs and entire cells. Plants, thanks to secondary metabolites like phenols and flavonoids, possess antimicrobial and antioxidant properties that protect cells from oxidative damage. Some plants can transform Ag^+^ into Ag°, resulting in nanoparticles with antioxidant characteristics (Duthie et al., 2003). AgNPs exhibit inhibitory effects on gene expression related to bacterial motility and biofilm formation, which plays a significant role in protecting bacteria from antibiotics (Barabadi et al., 2021; Talank et al., 2022). Consequently, researchers have turned to the biosynthesis of nanoparticles using plants and microorganisms as biocompatible, environmentally friendly methods. In dentistry, investment in research on natural products has led to the discovery of materials with enhanced therapeutic properties. Propolis, used in the preparation of new plant‐based drugs, has been widely studied. However, studies on the properties of Iranian propolis are currently limited and incomplete, and at this time, no research has evaluated the antibacterial activity of AgNPs synthesized from Iranian propolis on periodontal pathogens. Thus, this study aimed to investigate the synthesis of AgNPs using propolis and their effect on the formation of the *P. gingivalis* bacterial biofilm, a key microorganism in the development and progression of periodontal disease. The results of this study indicate that AgNPs synthesized from propolis have anti‐biofilm properties against *P. gingivalis*. Additionally, different concentrations of AgNPs (0.1%, 0.3%, and 0.5%) were found to significantly inhibit the formation of *P. gingivalis* bacterial biofilms (*p* < .05). Increasing the AgNPs concentration was associated with a reduction in *P. gingivalis* bacterial biofilm formation. Therefore, AgNPs with a concentration of 0.5% or higher can effectively inhibit the growth of *P. gingivalis* bacterial biofilms. The antibacterial properties of AgNPs follow a concentration‐dependent pattern. Studies by Nakao et al. (2020) and Sung et al. (2019) have shown the positive effects of propolis treatment on patients with chronic periodontitis, which aligns with the findings of this study. Sundaram et al. (2019) reported the potential of nanoparticles derived from plant exosomes in preventing or treating chronic periodontitis. Yoshimasu et al. (2018) found that EEP increased the permeability of *P. gingivalis* cell membranes, leading to their destruction. The study by Nakajima et al. (2016) reported that Brazilian propolis could alleviate glucose and fat metabolism disorders due to periodontitis and mitigate the effects of *P. gingivalis*. Matei et al. (2015) demonstrated that a mixture of chitosan, propolis extract, and AgNPs exhibited stronger inhibitory activity against *Diplodia seriata* mycelia compared to chitosan alone, supporting the growth‐inhibiting effect of AgNPs. Skaba et al. (2013) showed that toothpaste enriched with Brazilian propolis extract displayed time‐dependent antimicrobial action, especially against gram‐positive bacteria, effectively removing dental plaque and improving the condition of the marginal periodontium, in line with the present study. Coutinho (2012) found that subgingival irrigation with propolis extract reduced sites infected with *P. gingivalis*, increasing the number of sites showing bleeding on probing, corroborating the present study's results. Halkai et al. (2017) demonstrated that AgNPs had antibacterial effects similar to chlorhexidine and ampicillin without causing bacterial side effects or resistance. Kaur et al. (2019) studied the antibacterial effect of a synergistic combination of vancomycin and AgNPs against both gram‐positive *S. aureus* and gram‐negative *E. coli*, showing increased antibacterial efficacy against both bacteria types. Holden et al. (2016) found that silver/gold nanoparticles coated with glutathione oxide inhibited the planktonic growth of *P. gingivalis* W83, an effect enhanced by hydrogen peroxide, similar to the conditions of oxidative stress within the periodontal pocket during chronic inflammation. Furthermore, Lu et al. (2013) demonstrated that the antibacterial activity of AgNPs against oral cavity pathogenic bacteria is size‐dependent, with 5 nm AgNPs exhibiting the highest antibacterial activity. Sivolella et al. (2012) evaluated the effects of AgNPs on bone surgery and found that they could be used to reduce bacterial adhesion, prevent biofilm formation, and delay peri‐implantitis due to their antimicrobial properties.

## 4 CONCLUSIONS

In conclusion, in this study, the hydroalcoholic extract of propolis was used for the synthesis of AgNPs. The results of this study showed that AgNPs synthesized from Iranian propolis significantly reduce the *P. gingivalis* bacteria biofilm formation. The antibiofilm activity increases significantly with the increasing concentration of AgNPs. The antibiofilm activity of synthesized AgNPs significantly increases in a dose‐dependent manner. The results of this study indicate that the use of AgNPs synthesized from propolis can be used to achieve better therapeutic results in the treatment of periodontal disease.

## AUTHOR CONTRIBUTIONS

Fahime Sadat Hashemiyan, Mohammad Yusef Alikhani, and Abbas Farmany performed the experiments. Morad Hedayatipanah and Abbas Farmany supervised the study. All authors read and approved the final manuscript.

## CONFLICT OF INTEREST STATEMENT

The authors declare no conflict of interest.

## Data Availability

Data supporting this study are included in the article.
